# Supplementary material for: Disentangling metabolic functions of bacteria in the honey bee gut
Source: PLoS Biol. 2017 Dec 12;15(12):e2003467. doi: 10.1371/journal.pbio.2003467 (PMC5726620; doi:10.1371/journal.pbio.2003467)
Supplement: S8 Data — (ZIP) [file pbio.2003467.s008.zip › Z-score_plots/954 microbial substrate dTDP-4-amino-4,6-dideoxy-5-C-methyl-L-mannose.pdf]

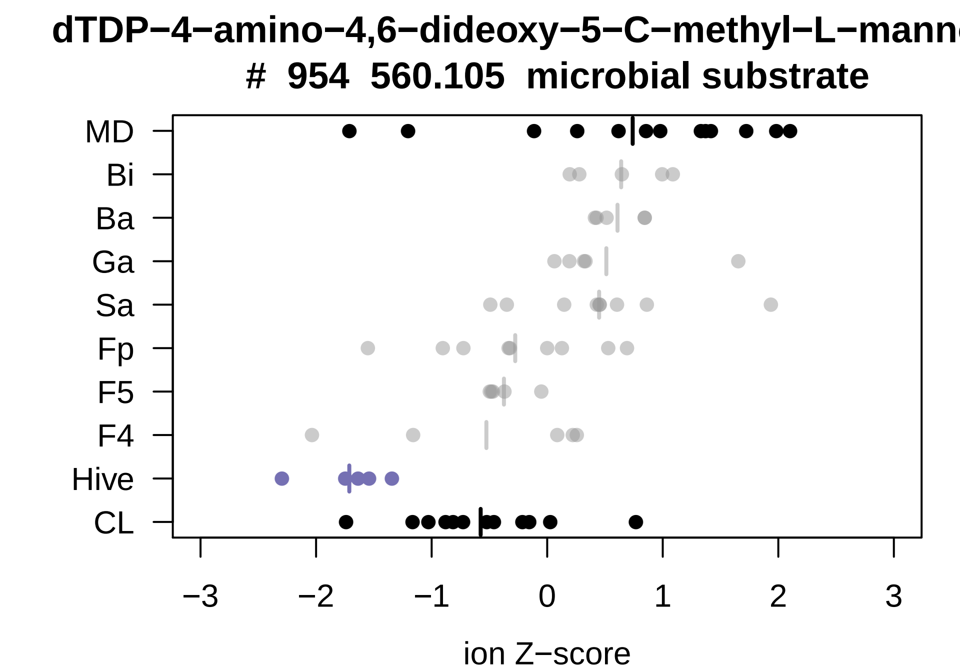
dTDP−4−amino−4,6−dideoxy−5−C−methyl−L−manno
954 560.105 microbial substrate
MD
Bi
Ba
Ga
Sa
Fp
F5
F4
Hive
CL
−3
−2
−1
0
1
2
3
ion Z−score
